# A Rare Case of Left Inguinoscrotal Hernia Containing Stomach

**DOI:** 10.7759/cureus.30838

**Published:** 2022-10-29

**Authors:** Karlbuto Alexandre, Zachary Vandeveer, John M Barnwell

**Affiliations:** 1 Department of Surgery, Detroit Medical Center (DMC) Sinai-Grace Hospital, Detroit, USA; 2 Medicine, University of Medicine and Health Sciences, Basseterre, KNA; 3 Surgery, Detroit Medical Center - Sinai Grace Hospital, Detroit, Michigan, USA

**Keywords:** incarcerated hernia, stomach, inguinal hernia repair, emergency general surgery, acute care surgery and trauma

## Abstract

This is the case of a 71-year-old male who presented to the emergency department with the chief complaint of left inguinoscrotal swelling and pain. The patient stated that he had nausea, vomiting, and constipation for a few weeks prior to the presentation. He also reported that he had a reducible, asymptomatic left inguinal hernia for the past 20 years. He began to experience pain in the left groin related to the hernia recently. During the past two weeks, he was having liquid bowel movements, and his last bowel movement occurred the morning of presentation. The patient did not report any fevers, chills, shortness of breath, or chest pain. His physical examination was remarkable for left lower quadrant fullness and mild abdominal distension. A large incarcerated left inguinoscrotal hernia was present, which markedly displaced and engulfed his penis. The patient was taken to the operating room for open inguinal hernia repair with mesh, where stomach and small bowel were encountered within the hernia sac. There was no ischemia noted, thus we repaired the hernia with mesh. The patient tolerated the procedure well and progressed postoperatively without incident. He was successfully discharged on postoperative day one. This case and literary review is a reference to the practicing general surgeon treating an incarcerated hernia containing the stomach.

## Introduction

Inguinal hernias occur commonly throughout the United States. On average, there are 800,000 hernia repairs annually in the United States [1-4]. Inguinal hernias are the most common abdominal type of hernias accounting for approximately 75% of all hernias. Almost one-third of males are diagnosed with an inguinal hernia during their lifetime. The highest incidence in adults is after 50 years of age [5]. Inguinal hernias can commonly involve the omentum, small bowel, colon, appendix, and bladder. However, an inguinal hernia containing the stomach is a rare occurrence [4-11]. Currently, most of the literature surrounding this topic has been limited to case reports and small case series [4-11]. This case report outlines the clinical presentation and management of a 71-year-old male, who was found to have a large uncomplicated, incarcerated inguinoscrotal hernia containing the stomach. This case and accompanying literary review serves as an excellent review for surgeons performing inguinal hernia repairs in the acute setting.

## Case presentation

**Figure 1:**
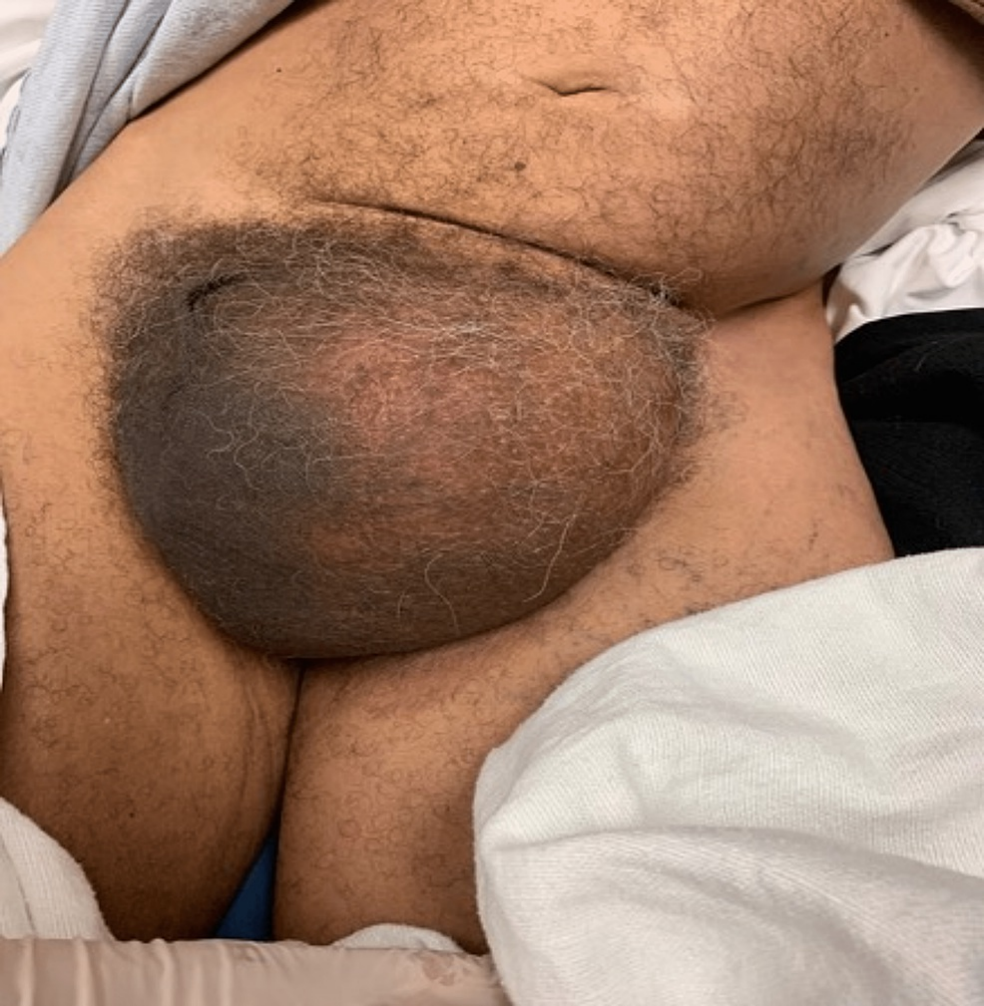
Large left inguinoscrotal hernia engulfing penis.

The patient is a 71-year-old male who presented to the emergency department with a chief complaint of a left inguinoscrotal hernia with associated pain, nausea, vomiting, and constipation. The patient stated that he had the hernia for the past 20 years, but over the past two weeks, it had doubled in size and become symptomatic. The patient also stated that over the two-week timespan the hernia became incarcerated as well and his bowel movements became mostly liquid. The patient's last bowel movement was on the morning of our initial evaluation. He did not admit to having any fevers, chills, shortness of breath, rectal bleeding, or chest pain. The patient was tachycardic with a heart rate of 100-120 bpm, systolic blood pressure was between 100s and 150s mmHg. The physical examination was remarkable for left lower quadrant fullness and mild abdominal distension from baseline, as well as a large incarcerated left inguinoscrotal hernia, engulfing the penis (Figure 1). There was no overlying skin change. The patient had a white blood cell count of 22.1 × 10^3 ^/μL, hemoglobin was 16.9 g, potassium was 2.4 mEq/L, blood urea nitrogen was 29 mg/dL, and creatinine was 2.69 mg/dL. Prior to sending the patient for computed tomography of the abdomen and pelvis with IV contrast, he was reassessed and found to be vomiting copious amounts of coffee ground emesis. A nasogastric tube was placed and put on low intermittent suction, approximately 500cc of gastric content was suctioned out. The patient was then sent for a CT scan.

**Figure 2:**
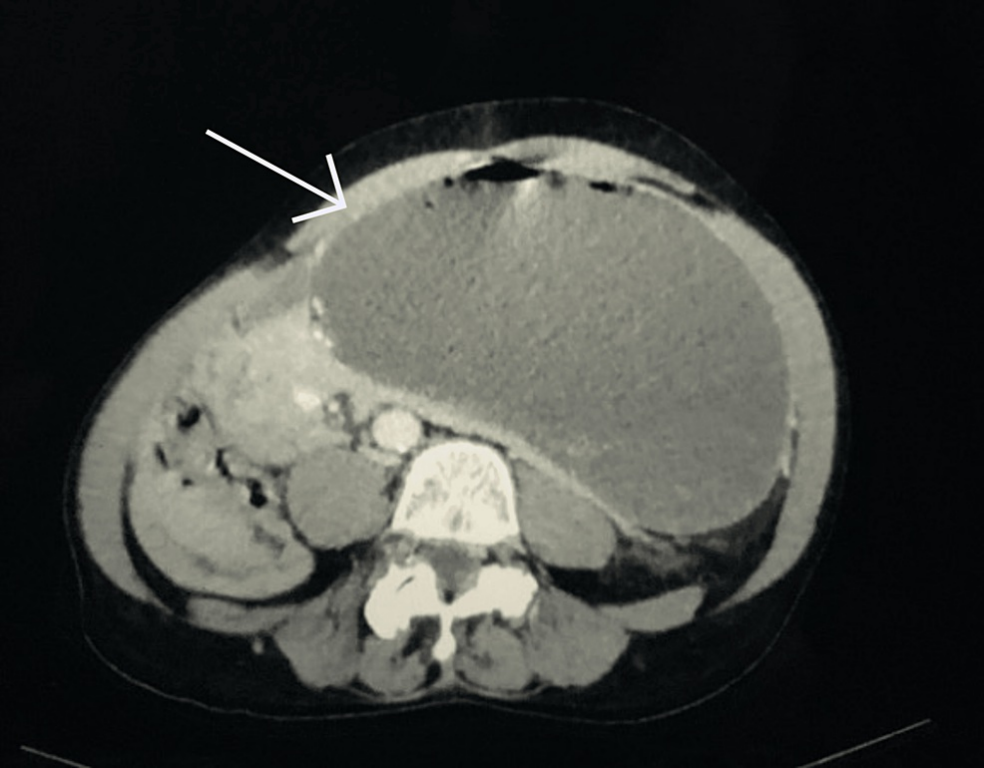
Computed tomography of abdomen and pelvis showing dilated stomach extending into the low pelvis. Arrow pointing to the dilated stomach extending into the low pelvis.

**Figure 3:**
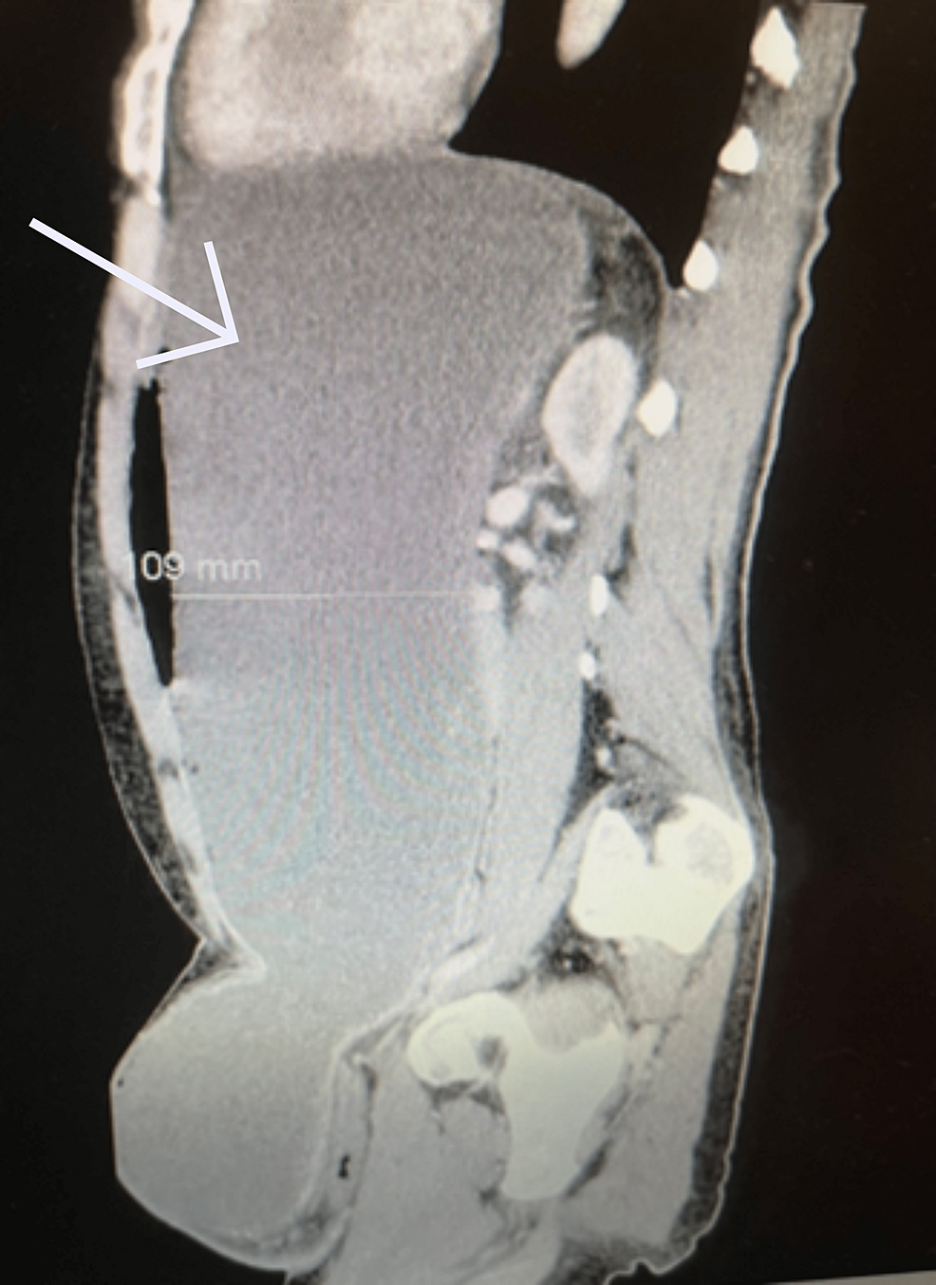
Computed tomography of abdomen and pelvis sagittal cross section showing dilated stomach extending into the left inguinal hernia. Arrow pointing toward dilated stomach measuring 109 mm in depth.

**Figure 4:**
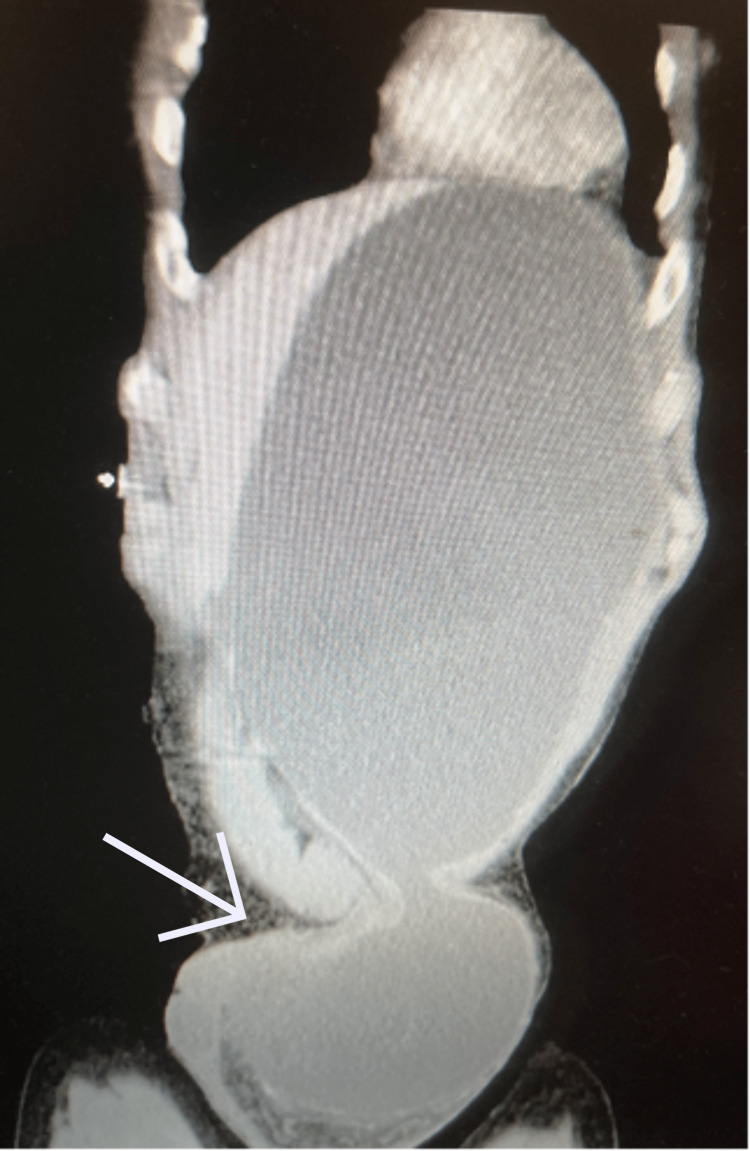
Computed tomography of abdomen and pelvis coronal cross section showing dilated stomach extending into the left inguinal hernia. Arrow pointing to incarcerated left inguinal hernia containing stomach.

**Figure 5:**
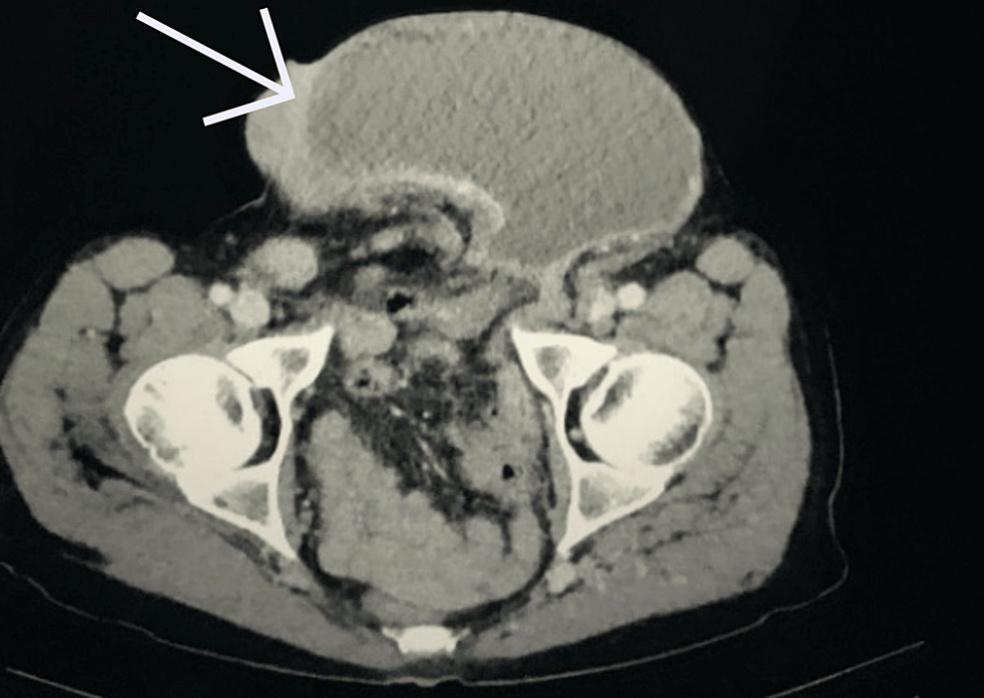
Computed tomography of abdomen and pelvis showing dilated stomach within left inguinal hernia sac. Arrow pointing toward stomach within left inguinal canal.

The results of the CT scan demonstrated a left inguinal hernia containing small bowel loops and a distended stomach without evidence of infarction. Gastric outlet obstruction was evident due to the antrum being compressed within the inguinal canal (Figures 2-5). The patient was subsequently resuscitated with aggressive intravenous fluids and broad-spectrum antibiotics. He was boarded and consented for an open left inguinal hernia repair with possible exploratory laparotomy. The left groin was explored via an oblique incision. A standard dissection was performed to explore the inguinal canal. Once the inguinal canal was exposed, the ilioinguinal nerve was identified and sacrificed proximally, and the iliohypogastric nerve was identified and spared. After dividing the cremasteric fibers, a large hernia sac was encountered and dissected from the cord. The sac was entered, and its contents included serous fluid, viable small bowel, and stomach. No obvious ischemia was noted. The excess sac was excised after reducing its contents and then closed with a purse string stitch using 3-0 Vicryl. An Ultrapro Hernia System-Large mesh (Ethicon Inc., Cincinnati, OH) was used to repair the hernia in a standard fashion. After completing the repair of the hernia, the skin was closed with a running 4-0 subcuticular stitch.

**Figure 6:**
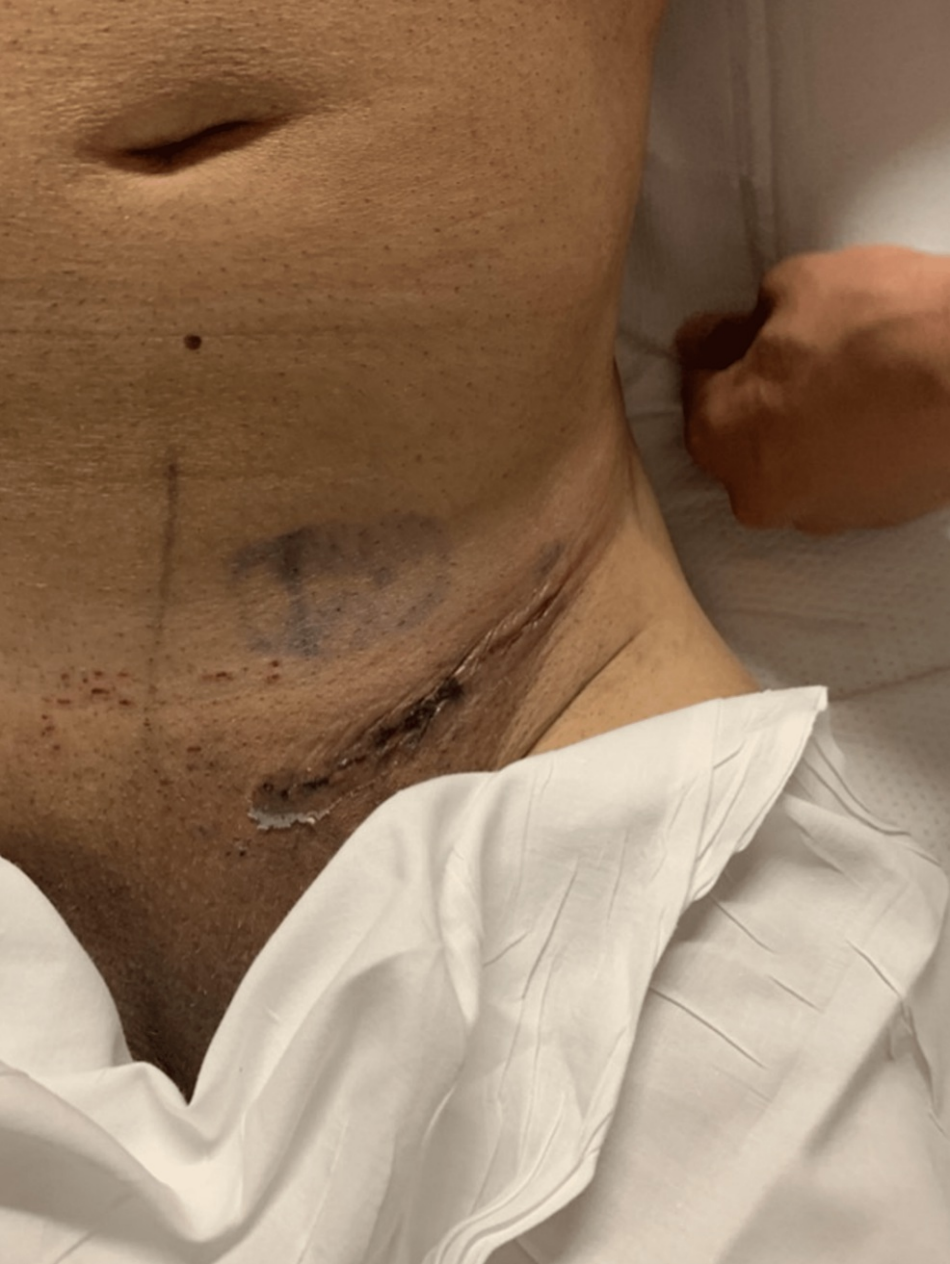
Image showing the final incision after surgery on postoperative day one.

The patient did not experience any postoperative complications. He was hospitalized after the procedure. His WBC normalized postoperatively. The patient had bowel function and the ability to tolerate an oral diet within 24 h. His incision was clean, dry, and intact as indicated in Figure 6. He was thus able to be discharged on postoperative day one.

## Discussion

Inguinal hernias can contain, small bowel, colon, and appendix, and involve urinary bladder. Those containing the stomach are extremely rare. This is probably due to the stomach being tethered in the upper abdomen by the gastrohepatic, gastrocolic, gastrosplenic, and gastrophrenic ligaments [6-10]. It is thought that the long-standing traction on the omentum and its attachments may be responsible for the descent of the stomach into the hernia sac [6-11]. A case report by Creedon et al. describes a patient with an inguinal hernia containing the pylorus of the stomach, small bowel, and the left hemicolon [9]. According to the report, there was no concern for necrotic tissue or gross contamination [9]. Thus, the authors performed open left-sided inguinal hernia repair with a standard Lichtenstein technique, using a Vicryl mesh (9). The patient’s postoperative course was complicated by hospital-acquired pneumonia but overall recovered well and was eventually discharged home. In our case, we used a similar approach to the surgical management of the hernia. However, we used a nonabsorbable synthetic mesh, as there was no gross contamination or necrosis.

The surgical approach can vary based on the presence or absence of necrosis, gross contamination, and previous surgery. A recent systematic review of inguinal hernias containing the stomach performed by Heylen et al. found 21 cases in their review [12]. All cases were male, with a mean age of 71 years. In 19 of the 21 cases, the authors included their surgical management [12]. Every patient with gastric perforation had operative management, of which eight of the 10 patients had a combination of midline laparotomy with an additional groin incision for hernia repair [12]. One other patient was treated surgically with a midline laparotomy alone and one with laparoscopic repair. There also was a group of nine patients that presented with gastric outlet obstruction, five patients were treated conservatively and four were treated with operative management [12]. Three out of the four cases of operative management in this group involved repair via groin incision. In the other case, the patient underwent a procedure with combined groin incision and midline laparotomy [12]. Of the 21 cases found in the literature review, only one case was done laparoscopically [12]. In our case, we did not think it was necessary to perform a midline laparotomy in addition to the oblique groin incision because we were able to adequately assess the stomach and small bowel via the inguinal exploration. There was also no evidence of infection, necrosis, or gross contamination which further supported the use of the left groin approach.

Moreover, in Heylen et al.'s study, 20 of the total cases were performed using an open incisional approach. The cases varied in their operative management of hernia repair [11]. In some cases, a Bassini, Lichtenstein, or Wantz-Stoppa hernia repair technique was used [12]. Mesh was used in three cases, including two with gastric perforation; none of these cases reported mesh infection [12]. If our patient had a gastric perforation or ischemic bowel contained within the hernia sac, we would likely have chosen to perform a primary McVay or Bassini repair; however, there were no contraindications to using nonabsorbable synthetic mesh in our case.

Furthermore, in the study by Heylen et al., eight patients presented with an inguinal hernia containing a gastric perforation [12]. In two cases, a gastrectomy was performed with a gastrojejunostomy, while in the other case a primary suture repair was done. Overall, clinical judgment must be used in treating gastric perforation within an inguinal hernia sac. This would depend on the condition of the patient and the origin of the perforation. Various techniques can include wedge resection with primary closure or formal antrectomy [12]. There was not any evidence of gastric perforation in our case, so resection or primary repair was not needed [12].

Patients presenting with gastric outlet obstruction from an inguinal hernia can be treated conservatively or may require immediate surgery after resuscitation. Conservative therapy is acceptable when the hernia can be reduced and there is no associated evidence of perforation or ischemia. This may allow for more time to improve the patient’s general condition should it be required [12]. Should the patient have a complicated hernia or have incarceration then more immediate repair is required following emergent resuscitation. These patients frequently present with dehydration related to vomiting and inability to tolerate PO intake. It is imperative for these patients to be adequately resuscitated from an electrolyte and hydration standpoint, as these imbalances are not uncommon due to persistent vomiting. Interestingly, in Heylen et al.'s study, five patients were noted to have been managed conservatively who had gastric outlet obstruction (GOO). These patients were selectively chosen because they had no signs of perforation or hemodynamic instability [12]. Particularly age and comorbidity status played a part in the selection of these patients [12]. They performed nasogastric tube decompression, intravenous fluid resuscitation, and bowel rest as temporizing measures to be able to avoid an operation in the acute setting and eventually perform electively under more optimal circumstances [12].

In addition, Heylen et al. also compared the cases they found in their systematic review with regard to performing a one-stage vs two-stage approach to the operative management of inguinal hernia repairs containing gastric perforations [12]. In total, 11 out of 13 cases were managed with a one-stage operation and two were managed in a two-stage operation where initial repair of the gastric perforation was performed during the first operation and the hernia was later repaired on index admission approximately three months later [12]. The two-stage operative approach can be appropriate in the “damage control setting” to obtain source control and get the patient to the critical care unit in a timely fashion [12]. Also, placing a synthetic mesh in a contaminated field is not standard of care. A two-stage procedure allows for the use of mesh in a clean field that will improve final repair outcomes.

In our case, the patient was hemodynamically stable with no evidence of gastric perforation. The patient did have evidence of gastric outlet obstruction. He was resuscitated with intravenous fluids and nasogastric tube decompression and taken to the operating room emergently in a timely fashion. He remained stable intraoperatively and postoperatively. There were no contraindications to placing mesh, we did not see it necessary to perform laparotomy as we were able to assess the viability of the stomach and bowel through the hernia defect. Thus, we did not perform any operative measures to secure the stomach to the abdominal wall. Given the emergent setting that the patient presented in, we felt more comfortable addressing the pathology via open approach instead of laparoscopically or robotically. The patient did well postoperatively and was discharged late in the day on postoperative day one after tolerating full liquid diet.

## Conclusions

Overall, the occurrence of inguinal hernias is common; however, an inguinal hernia containing the stomach is a very rare occurrence. The presentation of these hernias is usually seen in the acute setting as most patients delay having surgery until the hernia has grown to a considerable size or the patient has gastric outlet obstruction, gastric perforation, incarceration, or strangulation of the hernia. This article serves as a review of the rare occurrence of incarcerated inguinal hernias containing the stomach. There is no true consensus on managing and operating on patients with inguinal hernias containing the stomach. This report provides a good resource of information to successfully manage this pathology if encountered in practice.
